# Effects of Basalt and Carbon Fillers on Fire Hazard, Thermal, and Mechanical Properties of EPDM Rubber Composites

**DOI:** 10.3390/ma14185245

**Published:** 2021-09-12

**Authors:** Przemysław Rybiński, Bartłomiej Syrek, Anna Marzec, Bolesław Szadkowski, Małgorzata Kuśmierek, Magdalena Śliwka-Kaszyńska, Ulugbek Zakirovich Mirkhodjaev

**Affiliations:** 1 Institute of Chemistry, The Jan Kochanowski University, 25-406 Kielce, Poland; bartlomiejsyrek@wp.pl; 2 Institute of Polymer and Dye Technology, Faculty of Chemistry, Lodz University of Technology, 90-924 Lodz, Poland; boleslaw.szadkowski@dokt.p.lodz.pl (B.S.); malgorzata.kusmierek@dokt.p.lodz.pl (M.K.); 3 Department of Organic Chemistry, Faculty of Chemistry, Gdansk University of Technology, 80-233 Gdansk, Poland; magdalena.sliwka-kaszynska@pg.edu.pl; 4 Department of Biophysics, National University of Uzbekistan, Tashkent 700174, Vuzgorodok, Uzbekistan; u.z.mirkhodjaev@gmail.com

**Keywords:** polymer composites, EPDM rubber, carbon fillers, mineral fillers, basalt, thermal properties, fire hazard

## Abstract

Due to growing restrictions on the use of halogenated flame retardant compounds, there is great research interest in the development of fillers that do not emit toxic compounds during thermal decomposition. Polymeric composite materials with reduced flammability are increasingly in demand. Here, we demonstrate that unmodified graphene and carbon nanotubes as well as basalt fibers or flakes can act as effective flame retardants in polymer composites. We also investigate the effects of mixtures of these carbon and mineral fillers on the thermal, mechanical, and rheological properties of EPDM rubber composites. The thermal properties of the EPDM vulcanizates were analyzed using the thermogravimetric method. Flammability was determined by pyrolysis combustion flow calorimetry (PCFC) and cone calorimetry.

## 1. Introduction

The number of fatalities resulting from fires has been rising steadily since 1950. This is due mainly to the increasing use in construction of materials containing chemical compounds in the form of adhesives, auxiliary agents, and, above all, plastics, to replace conventional materials such as wood and steel [1,2,3,4]. These modern construction materials burn faster and hotter, and produce more smoke, making fires more hazardous. One of the main priorities of materials engineering is therefore to develop non-flammable or at least less flammable composite materials. Reducing the flammability of polymers requires a thorough knowledge of the reaction mechanisms that occur during the thermal decomposition of flame-retardant composites. The thermal decomposition of a polymer is an endothermic process, which always requires some initial external energy. The amount of energy necessary to initiate the process of thermal decomposition and combustion must be greater than the energy of the covalent carbon-carbon bond in the main chain (in most polymers, the C-C bond energy is in the range of 200 to 400 kJ/mol) [5,6]. The process of thermal degradation of the polymer (chain scission) is determined by the following factors: the presence of oxygen atoms in the main chain, impurities (e.g., catalyst residues), chemical defects in the chain, and the presence of so-called weak bonds, especially at the end of the chain. The degradation and eventual destruction of the polymer may therefore occur according to the following reaction mechanisms [7]:

− The proton transfer mechanism: in this case, two stable molecules are formed, one of which has a reactive carbon-carbon double bond;
− The free radical mechanism: in this case, the reaction does not stop at the stage of a single chain scission, but is an autocatalytic process in which further chain fragmentation reactions, as well as antagonistic thermal crosslinking reactions, occur. Under aerobic conditions, low molecular weight degradation products are formed, such as carboxylic acids, alcohols, aldehydes, and ketones, which are in turn a source of reactive hydrogen and hydroxyl radicals.

At temperatures above 300 °C, thermal degradation of polymers occurs under anaerobic conditions [7]. This is because the rate of thermal decomposition (pyrolysis) is much higher than the rate of oxygen diffusion into the solid reaction zone. Gaseous products of pyrolysis (no oxygen decomposition) penetrate the surface of the solid phase by means of diffusion and convection processes. When mixed with oxygen, they provide a source of free, high-energy radicals. The ignition of gaseous destructs, determined primarily by the oxygen concentration in the reaction environment, results in an increase in the temperature of the boundary layer and thus improves the efficiency of the pyrolysis reaction. The next stages of the combustion process can therefore take place without an external heat source. 

Halogen compounds are among the most effective flame retardants [8,9,10]. Their action is associated with a significant reduction in the efficiency or even stoppage of the process of free radical combustion in the gas phase. Halogen radicals formed as a result of the decomposition of antipyretic compounds in reactions with high-energy hydrogen radicals or hydroxyl radicals form low-reactive or inert chemical individuals. Modification of the combustion process using halogen radicals leads to a decrease in the efficiency of exothermic reactions, and thus to a decrease in both the temperature of the flame and the size of the pyrolysis zone. This in turn decreases the formation of combustible destructs. Despite the high efficiency of halogen compounds, an undoubted disadvantage of their use is the high toxicity and corrosiveness of the halogen hydrogen that forms as a result of their decomposition. Thus, in spite of its high effectiveness, limiting the flammability of polymeric materials based on organophosphorus compounds in a synergic system with a carbon donor has certain disadvantages. Among the most important is the reduced thermal stability of the flame-retardant polymer, as the polymer degrades under the influence of the phosphoric acid that forms [11,12,13,14]. 

The flammability of polymeric materials can also be lowered using endothermally dehydrated compounds, including primarily magnesium hydroxide and aluminum hydroxide. As a result of their thermal decomposition, water vapor is emitted which, by diluting the gaseous products of polymer decomposition, reduces their flammability. The magnesium or aluminum oxides formed during decomposition have relatively high heat capacity, which further lowers the temperature of the polymer. Furthermore, the use of endothermic flame-retardant compounds results in a significant reduction in smoke emissions. A major disadvantage is that to obtain satisfactory flame retardancy (over 50 phr) large amounts of hydroxides are needed, which deteriorate the mechanical properties of the composites [15]. 

It is well known that the main parameter indicating the intensity of the combustion process is the heat release rate (HRR). A flame retardant system should have the lowest HRR possible [16]. The literature shows that carbon fillers, especially in the form of flat or cylindrical graphene layers (CNTs), can effectively reduce the flammability of polymeric materials, due to their low thermal conductivity [17,18,19,20]. Basalt, which is a natural filler of volcanic origin with high thermal stability up to T = 1200 °C, is also seen as a promising filler for strengthening and reducing the flammability of polymeric materials. Basalt can be introduced into a polymer matrix either in the form of cut basalt fibers or as so-called basalt flakes [21,22,23]. 

Ethylene-propylene diene (EPDM) rubber is commonly used in the cable and automotive industry. It is characterized by very good resistance to oxygen, ozone, and atmospheric conditions, very good resistance to high temperature and water vapor, good resistance to polar substances, good elasticity, and good electrical properties. The disadvantages of EPDM rubber include the low adhesive properties of raw blends, the formation of efflorescence on the vulcanizate surface, the limited miscibility with diene rubbers, unsatisfactory oil resistance, and unsatisfactory thermal stability and flammability, due to its low susceptibility to thermal crosslinking and cyclization reactions [24]. 

This paper presents a comprehensive study of the impact of adding carbon fillers in the form of carbon nanotubes or graphene, or basalt filler in the form of cut basalt fibers or basalt flakes, to EPDM rubber composites. We also looked at the effect of the mixtures of carbon and basalt filler on the properties of the EPDM rubber vulcanizates. We examined the rheological, mechanical, and thermal properties as well as the flammability of the EPDM rubber composites.

## 2. Materials and Methods

### 2.1. Materials

**Figure 1:**
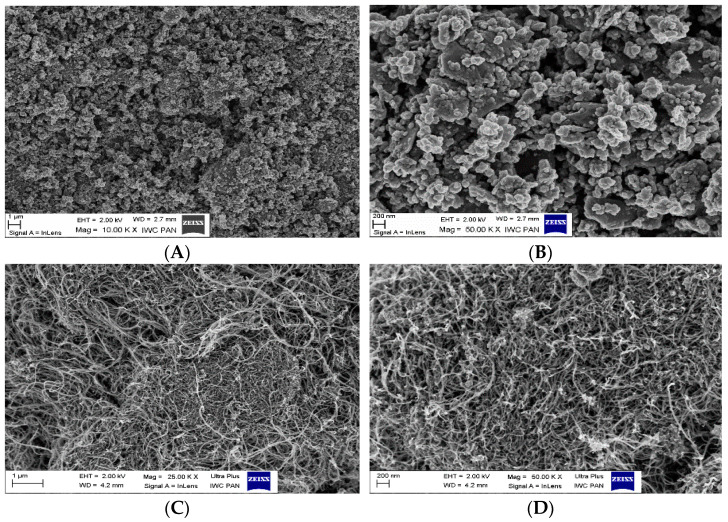

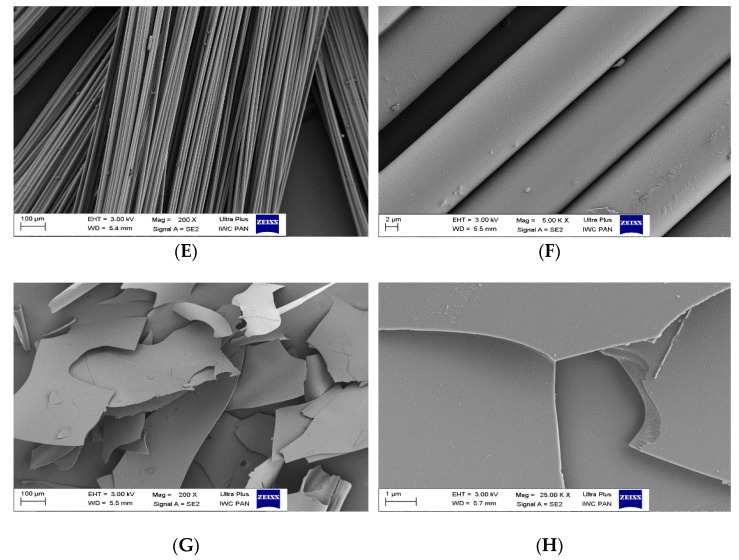
SEM photos: (**A**,**B**)—graphene flakes; (**C**,**D**)—carbon nanotubes; (**E**,**F**)—basalt fibers (BFS); (**G**,**H**)—basalt flakes (BFL).

“Keltan 21” (Mooney viscosity at 125 °C of 25, ethylene content of 60%) from Lanxess AG (Germany) was applied as an elastomer matrix. The crosslinking system consisted of sulfur from POCH (Gliwice, Poland), zinc oxide (ZnO) from Huta Oława (Oława, Poland), and N-cyclohexyl-2-benzotiazolilosulfeamide (Tioheksam CBS) from POCH (Gliwice, Poland). Carbon nanotubes (MWCNTs) and graphene were used as carbon fillers. MWCNTs with diameters of 8–15 nm, lengths of 10–50 µm, and specific surface areas greater than 110 m^2^/g were supplied by Cheap Tubes INC. (Cambridgeport, MA, USA). xGnP-C-500 Graphene nanoplatelets with a specific area of 500 m^2^/g were supplied by XG Sciences Inc (Lansing, MI, USA). Basalt flakes (BLF) from Tech Solutions (Skarżysko-Kamienna, Poland) and basalt fibers (BFS) were used as mineral fillers. BCS13-6.35-DRY with a length of 0.02 µm, width of 0.02 µm, and height of 0.022 µm were obtained from Basaltex (Wevelgem, Belgium) (Figure 1).

### 2.2. Methods

Elastomer blends were prepared using a laboratory mill with a roll length of 330 mm and a diameter of 140 mm. Each compound was mixed with friction of 1.1 at a temperature of 40 °C for approximately 15 min. The EPDM was first masticated for 3 min on the mill. The other ingredients, including the curing agents and fillers, were added successively to the EPDM matrix. The compositions of the EPDM composites are presented in Table 1.

After 24 h, the elastomer rubber mixes were subjected to rheometric measurements using a moving die rheometer model (Alpha Technologies, New York, NY, USA), according to the ISO 6502 standard. The curing process was performed using an electrically heated hydraulic press at 160 °C with 15 MPa of pressure for curing times consistent with the vulcanization parameters. As a result, a series of cured EPDM rubber composites was obtained, filled with different quantities of fillers. The mechanical properties of the composites were investigated using a universal strength machine (Zwick, Ulm, Germany) equipped with an extensometer. Tensile tests were performed on five dumbbell-shaped specimens of each composite at a crosshead speed of 500 mm/min, according to the ISO 37 standard. The tear resistance of the composites was measured in accordance with the ISO 34-1 standard for three trouser-shaped samples of each composite, at a test speed of 50 mm/min. The hardness of the composites was tested following the ISO48 standard, using a Shore A type digital microcomputer-controlled hardness tester (ZwickRoell, Ulm, Germany). The crosslink density of the EPDM vulcanizates was calculated on the basis of solvent-swelling measurements in toluene using the Flory–Rehner equation. The measurements were repeated four times for each composite. Thermal analysis (TGA, DTG) was performed using a thermal analyzer (Jupiter STA 449F3, Netzsch Company, Selb, Germany) in a temperature range of 25–700 °C, with a heating rate of 10 °C × min^−1^ in a nitrogen atmosphere. A cone calorimeter (Fire Testing Technology Ltd., East Grinstead, UK) was used to evaluate the flammability of the EPDM composites, according to the PN-ISO 5660 standard. Squared specimens with dimensions of 100 × 100 × 2 mm were irradiated horizontally using a 35 kW/m^2^ heat flux. 

The flammability of the EPDM composites was also tested, using a PCFC—pyrolysis combustion flow calorimeter (Fire Testing Technology Ltd., East Grinstead, UK). The temperature of the pyrolizer was 750 °C and the heat of the combustor was 900 °C. The following parameters were recorded: maximum heat emission rate (HRRmax), total heat emitted (HR), heat capacity. Each sample was heated using a linear temperature program. The volatile thermal degradation products were swept from the pyrolysis chamber by an inert gas and combined with excess oxygen in a tubular furnace at a temperature of 900 °C to force complete combustion (oxidation) of the fuel. The combustion products (CO_2_, H_2_O, and acid gases) were scrubbed from the gas stream. The transient heat release rate was calculated from the measured flow rate and oxygen concentration after correcting for flow dispersion. The maximum (peak) value of the PCFC heat release rate normalized for the initial sample mass and heating rate was used as a material flammability parameter, measured in units of heat release capacity (J/gK), which depends only on the chemical composition of the sample and is proportional to the burning rate of the material in a fire [25].

## 3. Results

### 3.1. Morphology of Composites

**Figure 2:**
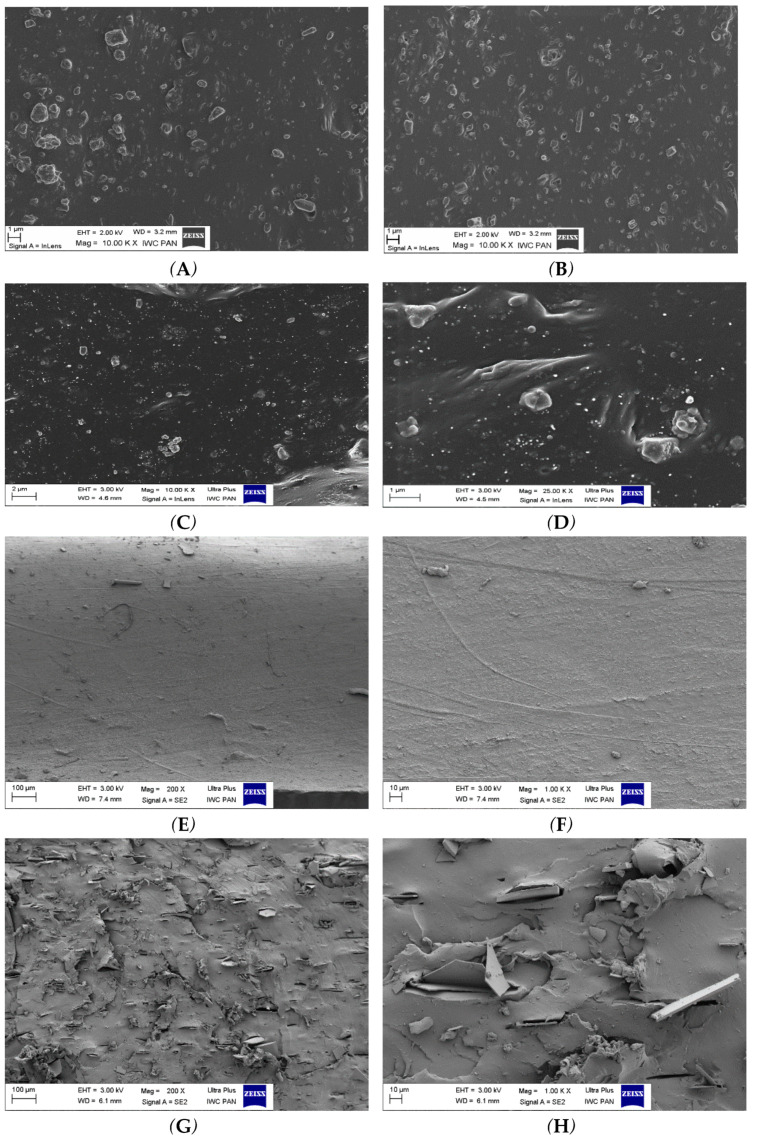

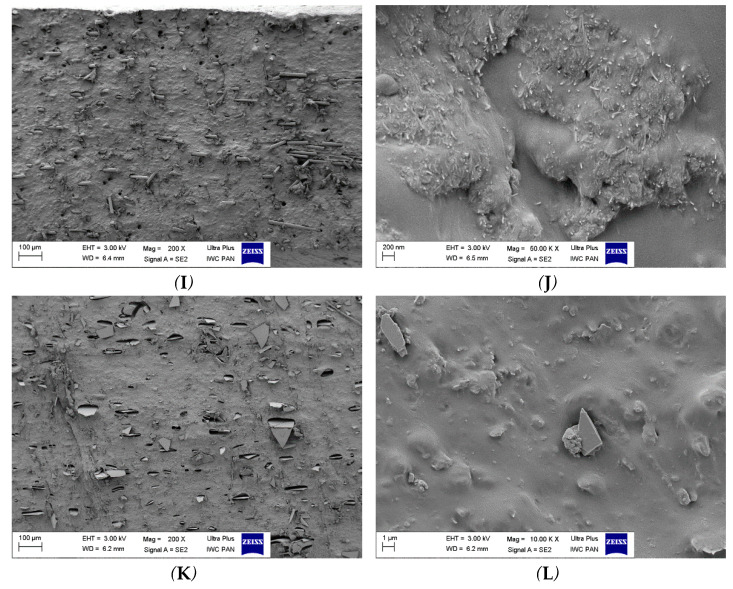
SEM photos of EPDM rubber composites. (**A**,**B**)—graphene flakes; (**C**,**D**)—carbon nanotubes; (**E**,**F**)—basalt fibers (BFS); (**G**,**H**)—basalt flakes (BFL); (**I**,**J**)—BFS and CNTs; (**K**,**L**)—graphene flakes and BFL.

The microstructures of the EPDM composites filled with carbon and mineral fillers were analyzed based on SEM photos. Well-dispersed mineral fillers should ensure the integrity of the boundary layer structure and provide carbon thermal insulation. Figure 2 presents SEM photos of the fractured surfaces of cured EPDM composites filled with the carbon filler, mineral filler, or carbon-mineral fillers. At the micron scale (Figure 2A–D), numerous GnP (graphene nanoparticles) and CNTs (carbon nanotubes) can be observed in the polymer matrix. Both the GnP and CNTs are uniformly distributed in the rubber matrix, which indicates good filler-polymer compatibility. The BFS and BFL mineral fillers are also uniformly distributed in the rubber matrix. As can be seen in Figure 2E–H, the dispersion of BFS in the rubber matrix was slightly better than the dispersion of BFL, which is a consequence of the larger size of the BFL in comparison to BFS. The BFL dispersed homogeneously in the polymer matrix, providing high thermal stability. Thanks to the lamellar structure, BLF very effectively inhibit the so-called channeling effect—i.e., the transport of mass and energy between the flame and the sample.

The BFS-CNT and BFL-graphene systems provided the most homogeneous boundary layer, which had a decisive effect on reducing degradation processes and the thermal destruction of the tested composites. 

### 3.2. Thermal Properties and Flammability Tests

**Figure 3:**
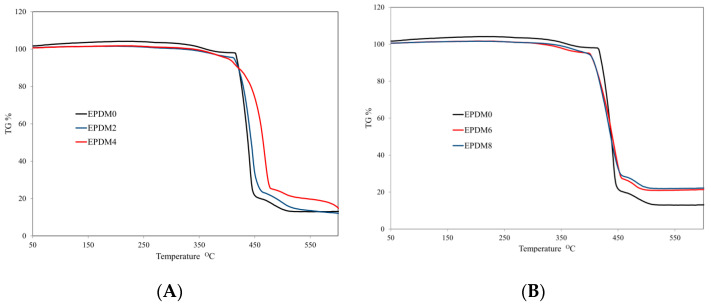

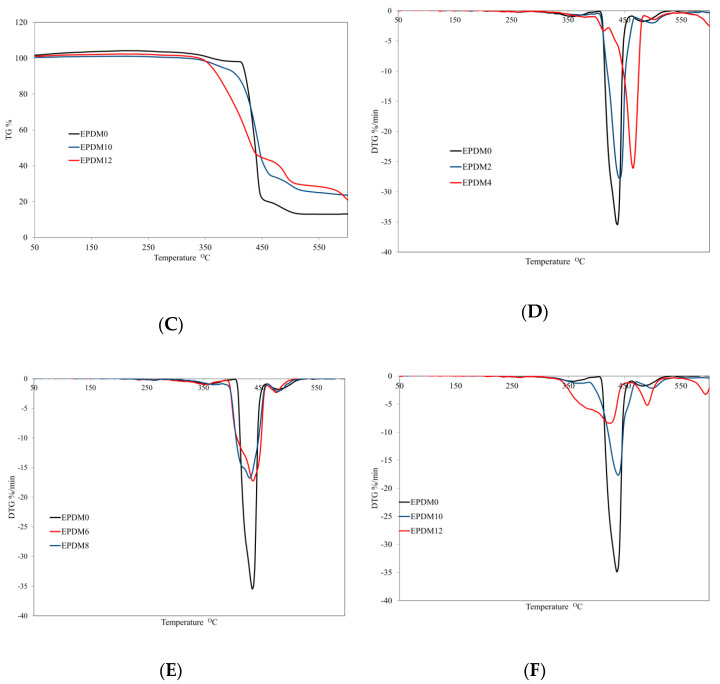
Thermogravimetric analysis data obtained for EPDM-filled composites: (**A**–**C**) TG curves and (**D**–**F**) DTG curves.

The addition of carbon fillers in the form of nonexpanding graphite (GnP) or carbon nanotubes (CNTs) did not affect the thermal transitions of the cured EPDM rubber. Both the unfilled and carbon-filled crosslinked EPDM rubbers were characterized by distinct one-stage thermal decomposition, occurring in the temperature range ΔT = 415–460 °C (Figure 3A,D).

The carbon fillers did not have an unequivocal impact on the thermal parameters of the composites. The graphene and carbon nanotubes caused hardly any change in 5% sample weight loss and temperature of onset of thermal decomposition (TR), but they caused an increase in T_50_ (50% sample weight loss) and T_RMAX_ (Table 2). Particularly significant increases in the values for T50 and T_RMAX_ was observed in the case of the vulcanizate containing carbon nanotubes, in comparison to the unfilled sample (EPDM0). The composite filled with 15 phr CNTs (EPDM4) was characterized by a 28 °C higher T_50_ parameter as well as a 29 °C higher value for T_RMAX_ in comparison to the reference composite (EPDM0). The improvement in thermal stability, expressed by the T_50_ and TR_MAX_ parameters, resulted from the high thermal conductivity of the carbon fillers, which enabled greater heat dissipation within the sample. Thus, the well-dispersed carbon fillers protected the polymer matrix from the external thermal radiation stream, as evidenced by an increase in the maximum temperature of thermal decomposition, T_RMAX_. Moreover, polymer chains located near carbon fillers are much slower to undergo thermal degradation processes, as measured by parameters T_50_ and T_RMAX_ [26,27] (Table 2).

An extremely important parameter from the point of view of thermal stability is the rate of thermal decomposition [28]. In our study, clear reductions in the value of the dm/dt parameter was observed in the materials containing graphene or carbon nanotubes (Figure 3A,D, Table 2). These decreases were related not only to the reduced segmental mobility of the polymer chains but also to the chemical properties of the carbon fillers. Graphene and carbon nanotubes, such as carbon black, are free radical scavengers [29,30]. Their presence in the elastomeric matrix therefore inhibits free radical reactions, while simultaneously increasing the probability of the recombination of primary macroradicals by prolonging their residence time in the cage. Moreover, both GnPs and CNTs are able form an internal three-dimensional spatial network, which increases the viscosity of liquid destructs. Because of solidification processes, the destructs form an insulating layer on the surface of the burning composite, which significantly reduces the rate of decomposition [7,14].

The value of the PR parameter (the residue after thermal decomposition) has a significant effect on the flammability of the composite. The higher the PR value, the less flammable gaseous debris enters the combustion zone, reducing the intensity of the combustion process. In the presence of carbon filler, not only did the value of the PR parameter increase, but the residue after thermal decomposition also showed a wider range of combustion temperatures. The initial value of the ΔTs parameter in the case of the EPDM4 composite was as much as 25 °C higher than that for the reference composite. This clearly indicates the insulating character of the residue of the samples containing carbon filler after combustion (Table 2). 

The incorporation of basalt filler into the matrix of EPDM rubber resulted in a significant reduction in its rate of thermal decomposition. The parameter dm/dt was as much as 51.3% lower in the case of the vulcanizate containing flake basalt (EPDM 6), and 52.7% lower in the case of the vulcanizate containing cut basalt fiber (Table 2, Figure 3B,E). The significantly lower dm/dt values for composites containing basalt filler compared to the unfilled sample (EPDM0) resulted both from polymer-filler interactions, including adsorption of polymer chains on the basalt surface, and from the high thermal capacity of basalt. Increased polymer-filler interactions result in a significant decrease in the segmental mobility of polymer chains and thus in the efficiency of degradation and chain transfer reactions. Moreover, by absorbing significant amounts of heat, basalt filler acts as a thermal shield that protects the composite from both degradation and destruction processes.

In the presence of basalt filler, the residue after thermal decomposition parameter (PR), as well as the residue at temperature T = 600 °C parameter (P_600_), increased significantly (Table 2). Thermally stable basalt, which does not undergo any thermal transformations, positively influenced the structure of the boundary layer formed during thermal decomposition and combustion, effectively impeding the mass and energy flow between the sample and the flame. The slightly higher value of the PR and P_600_ parameters for the EPDM 8 composite containing BFS (basalt fiber) compared to the EPDM 6 composite containing the same amount of BFS (basalt flakes) is due to the larger size and thus worse distribution of BFS relative to BFL in the polymer matrix (Figure 2E–H and Figure 3B,E). The EPDM12 composite containing both carbon nanotubes and chopped basalt fiber was characterized by the highest thermal stability. The drastic decrease in the value of parameter dm/dt, with a simultaneous increase in the values for parameters PR and P_600_, is associated with the formation of a homogeneous, insulating boundary layer (Table 2, Figure 3C,F). 

The increased thermal stability of the studied EPDM rubber composites reduced their flammability. Comparative analysis of the results of both PCFC (pyrolysis combustion flow calorimetry) and cone calorimetry clearly shows that the composites had lower flammability in comparison with the reference composite (EPDM0) (Parameters HRR and THR, Table 3).

The incorporation of the carbon fillers into the rubber (EPDM 1–4) and their rather homogeneous distribution in the polymer matrix resulted in the formation of a three-dimensional spatial network, which was responsible for increasing the viscosity of the liquid destructs as well as for limiting their diffusion velocity into the flame. Consequently, less heat was released in the combustion process (HRR and HRRMAX parameters) (Table 3 and Table 4). Graphene and carbon nanotubes are characterized by high thermal conductivity values ranging from 4.84 × 103 to 5.30 × 103 W/mK for graphene [31] and 2 × 103 W/mK for CNTs [32], respectively. The literature shows that the introduction of carbon fillers, especially graphene, into a polymer matrix increases the thermal conductivity coefficient of the composite, reducing its flammability. The high thermal conductivity of carbon fillers results in easier heat dissipation within the sample. Heat is distributed through the material and temperature increases at the surface are decelerated. Since both polymer decomposition and the release of volatile fuel are delayed, ignition of the polymer occurs later. The well-dispersed carbon fillers protect the polymer, especially in the first stage of thermal decomposition (before ignition) from the external thermal radiation stream, which is evidenced by an increase in the maximum temperature of thermal decomposition, T_RMAX_. In the presence of the carbon fillers, the AMLR parameter was considerably lower in the EPDM 2 and EPDM 4 samples, and their burning time was prolonged in comparison to the reference composite EPDM 0 (Table 4).

**Figure 4:**
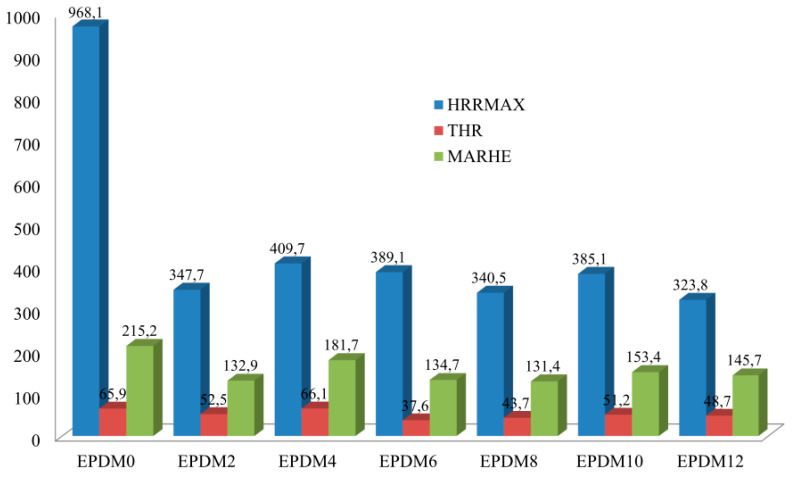
Flammability parameters of the EPDM-filler composites.

**Figure 5:**
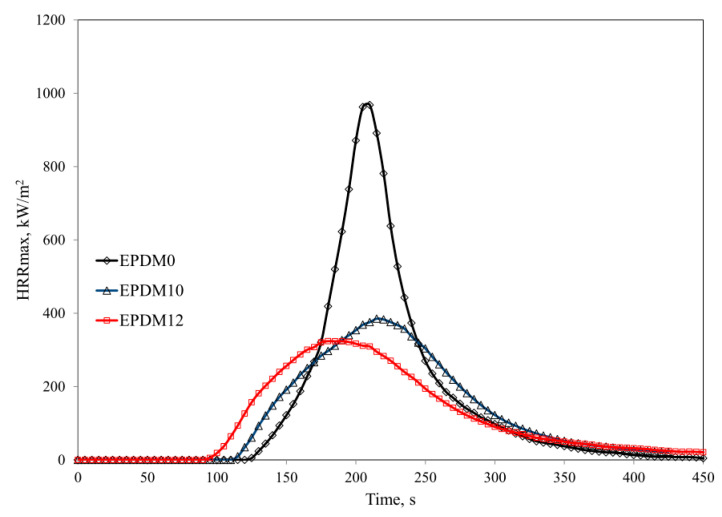
HRR curves of the EPDM-filled composites.

**Figure 6:**
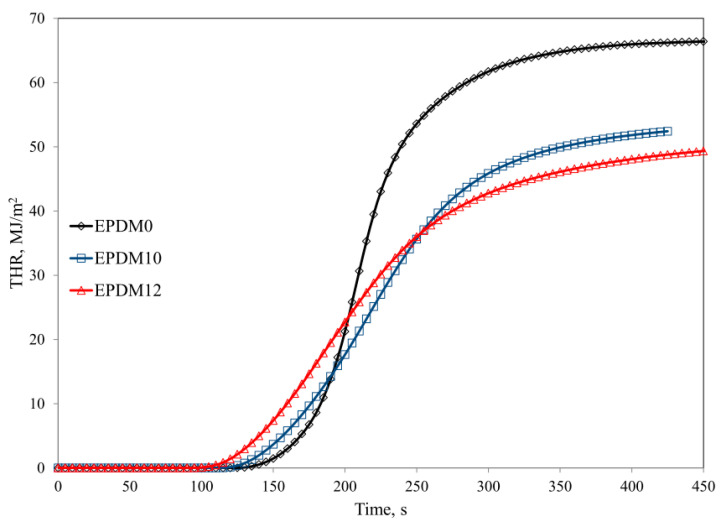
THR curves of the EPDM-filled composites.

The composites containing mineral fillers in the form of both BFL (EPDM 5–6) and chopped BFS (EPDM 7–8) were characterized by larger reductions in HRRMAX, THR, EHC, and EHCMAX values than the composites containing carbon fillers (Table 3 and Table 4, Figure 4, Figure 5 and Figure 6). The HRRMAX parameter, which is the key value illustrating combustion dynamics, was 59.8% lower in the case of the composite containing BFL (EPDM 6), whereas it was 64.8% lower in the case of the composite containing BFS (EPDM 8). The better performance of BFS over BFL is related to the better dispersion of BFS in the polymer matrix compared to BFL [33].

The mixture of CNTs and BFS was found to be effective system in terms of reducing the flammability of the crosslinked EPDM rubber. The significant reductions in HRR, HRRMAX, THR, EHC, AMLR and MARHE parameters observed for EPDM 11–12 were related to the formation of an insulating boundary layer that effectively limited mass and energy transport between the sample and the flame. The insulating properties of the boundary layer depend directly on the type of polymer, the amount of filler, and the degree of its dispersion in the polymer matrix. With an insufficient amount of filler and non-homogeneous distribution of the filler in the polymer matrix, the destructive products with relatively low viscosity that form during thermal decomposition of the nanocomposite can easily penetrate to the sample surface by convective transport. Due to the high temperature of the boundary layer, the liquid destructs form fast-growing blisters that push the filler particles away from each other, leading to the formation of so-called nanofiller islands that do not provide sufficient protection against the external heat source. Although EPDM rubber is characterized by low susceptibility to cyclization and thermal crosslinking processes, the fillers used in this study led to the formation of a homogeneous boundary layer, which effectively limited mass and energy transport between the sample and the flame (PR and P_600_ parameters, Table 1, Figure 5 and Figure 6). In the case of unfilled EPDM rubber, the boundary layer is formed of strongly degraded macromolecules and their liquid decomposition products. Because of the presence of the carbon filler, the boundary layer is more thermally stable and the polymer and carbon fillers from which it is formed are considerably less degraded (Table 2). Carbon fillers are an active sorbent of volatile products of destruction. The ability of carbon filler to adsorb volatile products increases with its specific surface (Table 4).

### 3.3. Rheometric and Crosslink Density Measurements

**Figure 7:**
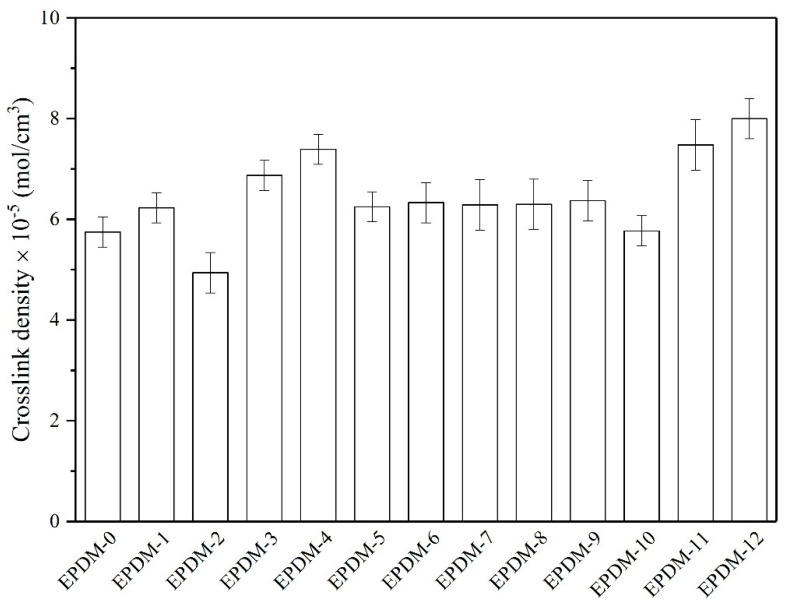
Crosslink density values of EPDM-filled composites.

To investigate the cure behavior of the EPDM rubber filled with different fillers, we performed rheometric and crosslink density measurements. The rheometric characteristics of the studied compositions are summarized in Table 5. In general, the torque values provide a qualitative overview of the dynamics the elastomer macromolecules during the curing process. As can be seen from the rheometric data, the minimum torques (M_min_) of the EPDM-filled compounds differed significantly, indicating alterations in their viscosity and processability. The most pronounced improvements in the minimum torque and increment of torque (ΔM) parameters were observed for samples filled with CNTs. These improvements can be explained by the high crosslink density and satisfactory interface adhesion between the CNTs and the EPDM matrix (Figure 7). Furthermore, the EPDM macromolecule chains were trapped in the CNT structure, reducing their mobility significantly. Thus, higher rheometric torque values were observed. This is in line with the results of previous studies on the use of fillers with tubelike structures to reinforce rubber composites [34,35]. On the other hand, the presence of GnP nanofiller resulted in EPDM compounds with lower ΔM and lower crosslink density (Table 5, Figure 7). The extended curing time (t_90_) and lower crosslink density of the EPDM/graphene composites resulted from the partial adsorption of curatives onto the outer surface of the graphene filler [36,37]. The addition of both type of basalt fillers did not affect significantly on the cure parameters (t_05_, t_90_, ΔM, M_min_) in comparison to reference sample. This fact can be explained by fact that the smooth and chemically inert surface of BFs results in the low interfacial adhesion between BFs and matrix, what was already reported in the literature [38].

The basalt fillers had no obvious influence on the crosslink density of the EPDM composites. The application of mixture composed of basalt fillers with carbon nanofillers resulted in a marked improvement in this parameter (Figure 7). This accelerating effect may be due to the very high thermal conductivity of GnP and CNT nanofillers, which favored heat transfer during the curing process [39].

### 3.4. Mechanical Performance

**Figure 8:**
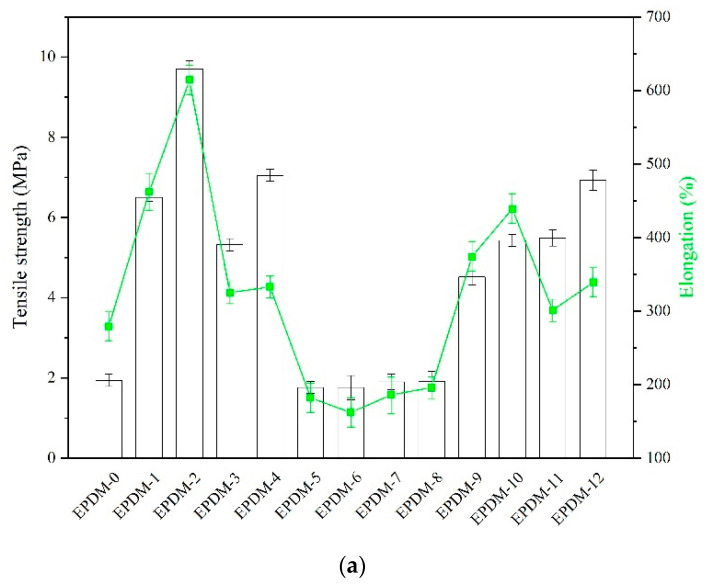

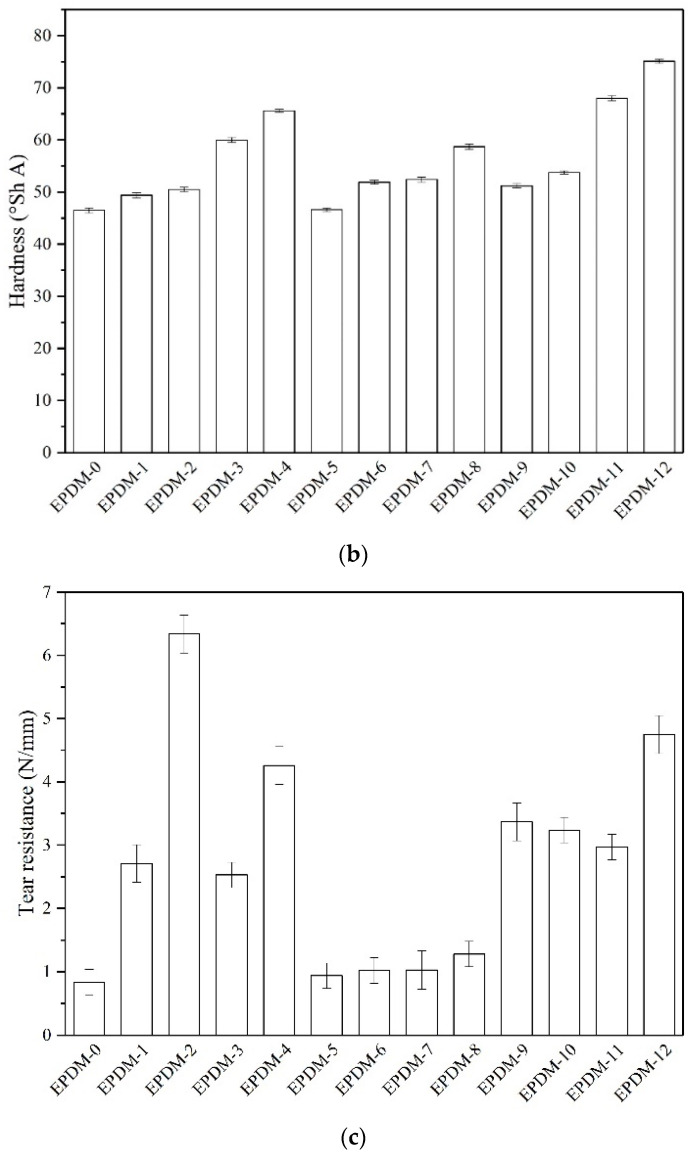
Mechanical performance of the EPDM composites: (**a**) tensile properties; (**b**) hardness; (**c**) tear resistance.

The mechanical performance (tensile strength, hardness, tear resistance) of the EPDM composites filled with the studied fillers is illustrated in Figure 8. As expected, the incorporation of graphene nanoplatelets and carbon nanotubes into the EPDM matrix resulted in a considerable improvement in the mechanical strength of the composite. These nanofillers are generally considered to be strongly reinforcing additives for elastomers [40,41]. In particular, increasing the concentration of these nanofillers up to 10 and 15 phr increased the tensile strength of the EPDM from 1.94 MPa to 6.50 Mpa and 9.70 Mpa for GnP-filled EPDM and 5.32 Mpa and 7.05 Mpa for MWCNTs-filled EPDM composites, respectively (Figure 8). These samples also showed significant improvements in elongation at break, which indicates the higher elasticity of these samples compared to the reference sample (EPDM-0). The considerable enhancement in mechanical performance following the incorporation of GnP and MWCNT into EPDM may be attributed to the large number of contact points between these nanofillers and the rubber macromolecules, as well as to their very high specific surface area [42]. On the other hand, neither the application of basalt fibers nor of basalt flakes led to visible changes in the tensile strength of EPDM. A similar effect was observed in our previous work on silicone rubber composites filled with differently structured basalt fillers [33]. The poor reinforcing activity of basalt fillers may be explained by their weak interfacial connection with the elastomer matrix. Li et al. [43] report that the smooth surface, lack of reactive groups, and low absolute connecting area of BFS may be responsible for its limited compatibility with the nitrile rubber matrix. The poor mechanical parameters of EPDM composites filled with BFS may be due to entanglement and disorientation of the fibers in the rubber matrix [44].

Nonetheless, the mixture made from basalt fillers and carbon nanofillers improved the tensile strength of the EPDM composites. The EPDM compositions containing 15 phr of basalt filler and 15 phr of GnP and/or MWCNT (EPDM-10 and EPDM-12) exhibited the most satisfactory tensile strength results, which were about 3.55 Mpa and 4.99 Mpa higher than the reference, respectively. Therefore, the mixture of the fillers containing carefully chosen ratios of basalt filler and GnP or MWCNT nanofillers may be an effective solution for reinforcing EPDM rubber. With increasing loads of both the fillers and individual basalt and carbon fillers, there was a gradual increase in the hardness of composites. The highest hardness values were noted for the EPDM-11 and EPDM-12 systems, with mixed fibrous fillers (MWCNTs and BFS). The improved hardness of the samples was most likely due to the enhanced crosslink density of the EPDM vulcanizates and good polymer-filler compatibility. The filler mixtures also showed very significant reinforcing activity during tear strength measurements. The tear resistance values of the EPDM composites improved with increasing amounts of the hybrid fillers. The highest tear resistance was reached for EPDM filled with 15 phr of graphene nanoplatelets. This trend is consistent with the previous tensile tests, indicating the superior mechanical reinforcement of GnP. Again, the presence of basalt fibers and flakes had a minor effect on tear resistance compared to the reference. The application of filler mixtures increased the tear strength, reaching a maximum of 4.75 N/mm for the EPDM-12 sample. This was most likely due to the presence of a high concentration (15 phr) of tubular-structure MWCNTs, which acted as crack-bridging elements in the hybrid systems, inhibiting tear propagation [45].

## 4. Conclusions

In this study, we investigated the thermal and mechanical properties as well as flammability of EPDM composites containing carbon fillers and basalt fillers. Based on the results, the following conclusions can be drawn:

- The carbon fillers were well-dispersed in the polymer matrix and protected the polymer from external thermal radiation, as evidenced by an increase in the maximum temperature of thermal decomposition, T_RMAX_.
- The introduction of basalt filler into the EPDM rubber matrix resulted in a reduction in the thermal decomposition rate.
- Thermally stable basalt, which does not undergo any thermal transitions, positively influenced the structure of the boundary layer formed during the thermal decomposition and combustion of the EPDM composites. The improved boundary layer effectively impeded the mass and energy flow between the sample and the flame.
- The EPDM12 composite containing both carbon nanotubes and chopped basalt fiber showed the highest thermal stability. The drastic decrease in the dm/dt value with a simultaneous increase in the parameters PR and P_600_ was associated with the formation of a homogeneous, insulating boundary layer.
- The most pronounced improvements in the minimum and increment of torque (ΔM) parameters were observed for the samples filled with MWCNT. These improvements can be explained by the high crosslink density of the composite and satisfactory interface adhesion between MWCNT and the EPDM matrix.
- Although basalt fillers had no obvious influence on the crosslink density of the EPDM composites, their application in mixture systems with carbon nanofillers resulted in a marked improvement in this parameter. Their accelerating effect may be due to the very high thermal conductivity of GnP and MWCNT nanofillers, which favored the process of heat transfer during curing.
- The incorporation of both graphene nanoplatelets and carbon nanotubes resulted in considerable improvements in the mechanical strength of the EPDM composites. The mixture made from basalt fillers and carbon nanofillers also improved the tensile strength of the EPDM composites.

## Figures and Tables

**Table 1:** Compositions of the EPDM composites.

Composition	Ingredients								
	EPDM	S	CBS	SA	ZnO	xGnP-C-500	MWCNTs	BFL	BFS
EPDM-0	100	1.5	1.5	1.0	5.0	–	–	–	–
EPDM-1	100	1.5	1.5	1.0	5.0	10.0	–	–	–
EPDM-2	100	1.5	1.5	1.0	5.0	15.0	–	–	–
EPDM-3	100	1.5	1.5	1.0	5.0	–	10.0	–	–
EPDM-4	100	1.5	1.5	1.0	5.0	–	15.0	–	–
EPDM-5	100	1.5	1.5	1.0	5.0	–	–	10.0	–
EPDM-6	100	1.5	1.5	1.0	5.0	–	–	15.0	–
EPDM-7	100	1.5	1.5	1.0	5.0	–	–	–	10.0
EPDM-8	100	1.5	1.5	1.0	5.0	–	–	–	15.0
EPDM-9	100	1.5	1.5	1.0	5.0	10.0	–	10.0	–
EPDM-10	100	1.5	1.5	1.0	5.0	15.0	–	15.0	–
EPDM-11	100	1.5	1.5	1.0	5.0	–	10.0	–	10.0
EPDM-12	100	1.5	1.5	1.0	5.0	–	15.0	–	15.0

EPDM—rubber, S—sulfur; CBS—N-cyclohexyl-2-benzotiazolilosulfeamide; SA—sterinic acid, XGnP-C-500—graphene, MWCNTs—carbon nanotubes, BFL—basalt flakes, BFS—basalt fibers.

**Table 2:** Thermal parameters of the EPDM rubber composites.

Sample	T_5_ (°C)	T_50_ (°C)	T_R_ (°C)	T_RMAX_ (°C)	dm/dt (%/min)	P_R_ (%)	ΔT_s_ (°C)	P_600_ (%)
EPDM-0	420	438	415	436	35.48	20.45	462–524	13.07
EPDM-1	413	440	415	438	29.57	22.17	465–523	12.65
EPDM-2	413	445	415	441	27.81	23.30	468–535	12.11
EPDM-3	395	450	415	451	24.20	24.70	472–525	14.41
EPDM-4	401	466	415	465	24.45	25.11	487–530	16.95
EPDM-5	403	439	405	436	21.85	22.90	462–514	17.01
EPDM-6	400	440	400	437	17.28	27.39	461–515	21.37
EPDM-7	395	437	400	435	16.29	29.12	463–520	22.80
EPDM-8	395	437	400	431	16.77	28.81	463–515	22.17
EPDM-9	410	450	410	440	18.03	25.78	477–530	17.29
EPDM-10	380	445	400	438	17.86	34.43	472–535	23.63
EPDM-11	378	460	360	449	13.36	44.07	475–530	34.13
EPDM-12	361	433	350	449	8.40	45.37	472–530	35.12

T_5_, T_50—_temperatures of 5% and 50% sample mass loss; T_R_—temperature of thermal decomposition; T_RMAX_—temperature of maximum rate of thermal decomposition; dm/dt—maximum rate of thermal decomposition; P_R_—residue after thermal decomposition; ΔTs—temperature range for combustion of residue; P600—residue after heating to T = 600 °C.

**Table 3:** Microcalorimeter analysis of the EPDM rubber composites.

Sample	HRR (W/g)	THHR (°C)	THR (kJ/g)	HRC (J/gK)
EPDM-0	1791	480	66.5	1811
EPDM-1	1441	480	58.7	1451
EPDM-2	1404	478	58,0	1410
EPDM-3	1478	480	61.8	1500
EPDM-4	1430	483	58.7	1436
EPDM-5	1412	484	56.9	1421
EPDM-6	1500	480	60.1	1507
EPDM-7	1345	480	59.2	1347
EPDM-8	1442	478	56.9	1469
EPDM-9	1426	479	57.0	1410
EPDM-10	1274	483	51.8	1277
EPDM-11	1263	482	49.7	1252
EPDM-12	1205	478	50.2	1194

HRR–heat release rate; THHR–temperature of heat release rate; THR–total heat release; HRC–heat capacity.

**Table 4:** Cone calorimetry flammability test results for EPDM composites.

Sample	t_i_ (s)	t_f-0_ (s)	HRR (kW/m^2^)	HRR_MAX_ (kW/m^2^)	tHRR_MAX_ (s)	THR (MJ/m^2^)	EHC (MJ/kg)	EHC_MAX_ (MJ/kg)	AMLR (g/m^2^×s)	FIGRA (kW/m^2^×s)	MARHE (kW/m^2^)
**EPDM0**	104	398	222.5	968.1	210	65.9	36.2	79.3	12.56	4.61	215.2
**EPDM1**	144	523	152.1	425.9	250	57.7	33.8	69.4	5.06	1.70	137.7
**EPDM2**	143	503	145.9	347.7	240	52.5	30.2	79.6	4.96	1.44	132.9
**EPDM3**	96	434	191.4	442.4	200	64.0	34.1	78.1	6.05	2.21	191.5
**EPDM4**	106	447	194.1	409.7	205	66.1	35.4	72.6	3.38	1.99	181.7
**EPDM5**	117	330	176.7	399.9	185	37.1	22.2	70.9	13.38	2.16	129.3
**EPDM6**	113	327	175.3	389.1	180	37.6	23.5	60.5	14.28	2.16	134.7
**EPDM7**	102	427	142.8	368.1	175	46.6	29.3	63.5	8.76	2.10	148.3
**EPDM8**	113	380	164.5	340.5	190	43.7	28.3	73.3	9.46	1.79	131.4
**EPDM9**	125	378	191.1	399.5	210	48.6	30.2	79.8	10.72	1.90	145.9
**EPDM10**	110	382	186.7	385.1	215	51.2	31.0	72.8	10.59	1.79	153.4
**EPDM11**	107	457	143.7	320.7	225	50.2	28.9	79.8	7.74	1.42	135.2
**EPDM12**	85	421	143.3	323.8	180	48.7	29.1	72.9	8.22	1.79	145.7

t_i_—time to ignition; t_f-0_—time to flameout; HRR—heat release rate; HRR_MAX_—maximum heat release rate; tHRR_MAX_—time to maximum heat release rate; THR—total heat release; EHC—effective heat of combustion; EHC_MAX_—maximum effective heat of combustion; AMLR—average mass loss rate; FIGRA—HRR_MAX_/tHRR_MAX_; MARHE—maximum average heat of emission.

**Table 5:** Rheometric properties of the EPDM-filled composites.

Sample	t_05_ (min)	t_90_ (min)	M_min_ (dNm)	ΔM (dNm)
EPDM-0	12.92	28.75	0.86	13.69
EPDM-1	2.95	35.68	1.28	13.67
EPDM-2	2.44	41.31	1.66	13.12
EPDM-3	5.71	22.92	2.08	22.03
EPDM-4	4.63	23.67	2.99	26.32
EPDM-5	14.25	30.73	0.99	15.96
EPDM-6	13.91	30.02	1.02	16.66
EPDM-7	13.60	29.38	0.89	14.68
EPDM-8	13.23	33.39	0.95	16.52
EPDM-9	2.87	34.56	1.45	16.19
EPDM-10	2.41	42.40	1.88	13.69
EPDM-11	5.35	22.49	2.15	23.77
EPDM-12	4.77	24.28	3.38	28.26

t_05_—scorch time; t_90_—curing time, M_min_—minimum torque; ΔM—increment torque.

## Data Availability

Data sharing not applicable.
